# Exploring the Role of Plasma Lipids and Statin Interventions on Multiple Sclerosis Risk and Severity: A Mendelian Randomization Study

**DOI:** 10.1212/WNL.0000000000207777

**Published:** 2023-10-24

**Authors:** Mona M. Almramhi, Chris Finan, Catherine S. Storm, Amand F. Schmidt, Demis A. Kia, Rachel Coneys, Sandesh Chopade, Aroon D. Hingorani, Nick W. Wood

**Affiliations:** From the Department of Clinical and Movement Neurosciences (M.M.A., C.S.S., D.A.K., R.R.C., N.W.W.), University College London Queen Square Institute of Neurology, United Kingdom; Department of Medical Technology (M.M.A.), Faculty of Applied Medical Sciences, King Abdulaziz University, Jeddah, Kingdom of Saudi Arabia; Institute of Cardiovascular Science (C.F., A.F.S., S.C., A.D.H.), Faculty of Population Health, and Health Data Research UK London (A.D.H.), University College London; British Heart Foundation University College London Research Accelerator (C.F., A.F.S., S.C., A.D.H.), United Kingdom; and Department of Cardiology (C.F., A.F.S.), Division Heart and Lungs, University Medical Center Utrecht, the Netherlands.

## Abstract

**Background and Objectives:**

There has been considerable interest in statins because of their pleiotropic effects beyond their lipid-lowering properties. Many of these pleiotropic effects are predominantly ascribed to Rho small guanosine triphosphatases (Rho GTPases) proteins. We aimed to genetically investigate the role of lipids and statin interventions on multiple sclerosis (MS) risk and severity.

**Method:**

We used two-sample Mendelian randomization (MR) to investigate (1) the causal role of genetically mimic both cholesterol-dependent (through low-density lipoprotein cholesterol (LDL-C) and cholesterol biosynthesis pathway) and cholesterol-independent (through Rho GTPases) effects of statins on MS risk and MS severity, (2) the causal link between lipids (high-density lipoprotein cholesterol [HDL-C] and triglycerides [TG]) levels and MS risk and severity, and (3) the reverse causation between lipid fractions and MS risk. We used summary statistics from the Global Lipids Genetics Consortium (GLGC), eQTLGen Consortium, and the International MS Genetics Consortium (IMSGC) for lipids, expression quantitative trait loci, and MS, respectively (GLGC: n = 188,577; eQTLGen: n = 31,684; IMSGC (MS risk): n = 41,505; IMSGC (MS severity): n = 7,069).

**Results:**

The results of MR using the inverse-variance weighted method show that genetically predicted *RAC2*, a member of cholesterol-independent pathway (OR 0.86 [95% CI 0.78–0.95], *p*-value 3.80E-03), is implicated causally in reducing MS risk. We found no evidence for the causal role of LDL-C and the member of cholesterol biosynthesis pathway on MS risk. The MR results also show that lifelong higher HDL-C (OR 1.14 [95% CI 1.04–1.26], *p*-value 7.94E-03) increases MS risk but TG was not. Furthermore, we found no evidence for the causal role of lipids and genetically mimicked statins on MS severity. There is no evidence of reverse causation between MS risk and lipids.

**Discussion:**

Evidence from this study suggests that *RAC2* is a genetic modifier of MS risk. Because *RAC2* has been reported to mediate some of the pleiotropic effects of statins, we suggest that statins may reduce MS risk through a cholesterol-independent pathway (that is, RAC2-related mechanism(s)). MR analyses also support a causal effect of HDL-C on MS risk.

## Introduction

Findings from the phase 2 MS-STAT trial (a randomized, placebo-controlled trial) showed that a high dose of simvastatin (80 mg per day) led to a significant drop in brain atrophy (by 43%) and disability progression among 140 patients with secondary progressive multiple sclerosis (MS) over 2 years.^1^ However, whether statins' beneficial effects on MS are mediated by cholesterol-lowering or cholesterol-independent pathway is not clear yet.

Indeed, recent evidence derived from clinical and experimental animal models of autoimmune diseases has shown that statins exert immunomodulatory and anti-inflammatory effects beyond their lipid-lowering properties that may be beneficial in autoimmune diseases such as MS.^2,3^ Many of these effects are predominantly ascribed to statins' capacity to inhibit the isoprenylation (also known as prenylation or lipidation) of Rho small guanosine triphosphatases (GTPases, also known as small G-proteins).^4-6^

**Figure 1:**
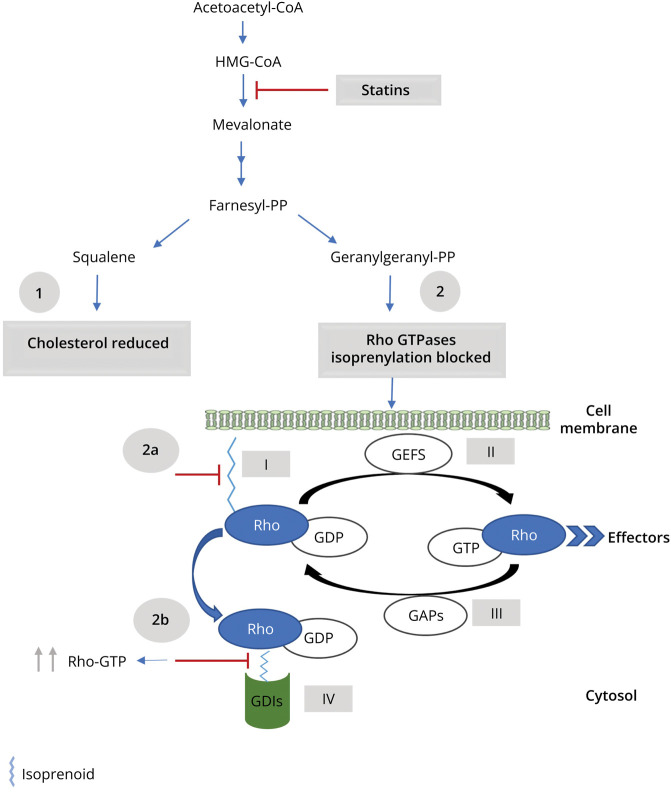
Statin Effects on Cholesterol and Rho GTPases *HMGCR* inhibition by statins leads to (1) reduction in the synthesis of cholesterol (2) and prevention of the synthesis of isoprenoids (such as Farnesyl-PP and Geranylgeranyl-PP). Isoprenoids are essential molecules for the prenylation and functioning of the Rho GTPase family.^10,50^ After isoprenylation, the Rho proteins localize to a target cell membrane (I) and are activated by GEFs that facilitate the exchange of GDP for GTP^10,50^ (II). This enables them to pass on signals to corresponding downstream effectors and regulate numerous cellular functions.^10,50^ Finally, the Rho proteins interact with GAPs that hydrolyze GTP to GDP, thereby inactivating the Rho proteins^10,50^ (III). When the Rho proteins are inactivated (GDP-bound form), GDIs extract them from the membrane and sequester the proteins in the GDP-bound form into the cytosol^10,50^ (IV). Thus, preventing the isoprenylation of Rho GTPases by statins lead to (2a) the inhibition of Rho protein translocation to the plasma membrane and prevents the activation of their downstream effectors^7^ (2b) and disruption of GDIs-Rho GTPase binding, which causes an increase in the levels of the cytosolic GTP-bound forms of Rho GTPases.^8,9^ GAPs, GTPase-activating proteins; GDIs, guanine nucleotide dissociation inhibitors; GEFs, guanine nucleotide exchange factors; HMGCR, 3-Hydroxy-3-Methylglutaryl-CoA Reductase; Rho GTPases, Rho small guanosine triphosphatases.

Statins exert effects through Rho GTPases by 2 distinct mechanisms: preventing Rho proteins from localizing to the membrane localization and loading Rho proteins with GTP (Figure 1). By inhibiting 3-hydroxy-3-methylglutaryl coenzyme A reductase (*HMGCR*), statins prevent the synthesis of isoprenoid intermediates and the subsequent isoprenylation of Rho GTPases.^7^ This leads to the inhibition of Rho protein translocation to the plasma membrane and thus prevents the activation of their downstream effectors.^7^ The second mechanism by which statins exert effects through Rho GTPases is GTP loading, which is the conversion of Rho proteins to their active form (GTP-bound). Inhibition of isoprenoid biosynthesis by statins results in disruption of guanine nucleotide dissociation inhibitors (GDIs)–Rho GTPase binding, which provides a potential mechanism for GTP loading of the cytosolic Rho proteins.^8,9^ GDIs are a negative regulator of Rho GTPases that only bind to isoprenylated Rho proteins to sequester them in the inactive form (GDP-bound) into the cytosol, preventing them from anchoring to membranes or being activated by guanine nucleotide exchange factors.^10^ Thus, in the absence of isoprenoid intermediates, GDIs cannot bind to Rho proteins, allowing them to be constitutively active (GTP-bound).^10^

A previous Mendelian randomization (MR) analysis used single-nucleotide polymorphisms (SNPs) within *HMGCR* gene region to mimic the effects of statins on the risk of MS developing through *HMGCR* inhibition.^11^ This study revealed no causal link between these SNPs and MS risk, suggesting that statins have no effect on MS risk.^11^ *HMGCR* is the target for statins; therefore, it is not surprising that MR studies focus on *HMGCR* to mimic the effects of statins. Nevertheless, by only targeting *HMGCR*, these studies examined the cholesterol-lowering effect only and may have missed observing the statins' pleiotropic effects. Furthermore, the effect of statins on MS severity has not yet been established. To address this knowledge gap, we adopted two-sample MR approach to genetically mimic both cholesterol-dependent and cholesterol-independent effects of statins to explore whether statins' effects on MS risk and/or MS severity, if any, are mediated by lowering cholesterol or are independent of cholesterol. In particular, the cholesterol-dependent pathway was studied by (a) examining the causal role of genetically predicted change in the blood expression levels of 25 genes (including the *HMGCR* gene) that encode proteins involved in cholesterol biosynthesis and (b) examining the causal role of genetically predicted LDL-C, given that LDL-C is a relevant prognostic factor for assessing the degree of *HMGCR* inhibition.^12^ The cholesterol-independent pathway was studied by examining the causal role of genetically predicted change in the blood expression levels of 20 genes that encode Rho GTPase family members. We sought also to examine the causal role of genetic predisposition to increased other major plasma lipid fractions (high-density lipoprotein cholesterol [HDL-C] and triglycerides [TG]) in MS risk and severity. In addition, the reverse causation between HDL-C, LDL-C, TG, and MS risk is addressed in this study. Because no single loci achieved genome-wide significance in MS severity data, we were unable to perform a reverse causation between lipid fractions and MS severity.

We tested 2 hypotheses to examine whether statins influence MS through cholesterol-dependent or cholesterol-independent pathways:

1. We would expect statins causally influence MS through lowering blood cholesterol levels if we obtain:
  - A statistically significant causal estimates for MR analyses involving LDL-associated SNPs.
  - A statistically significant causal estimates for MR analyses involving SNPs of HMGCR and any other downstream genes involved in cholesterol biosynthesis.
2. By contrast, we would expect statins causally influence MS through cholesterol-independent pathway, if we obtain a statistically significant causal estimates for MR analyses involving SNPs of Rho GTPases.

In simple terms, MR is a type of “instrumental variable” analysis that uses genetic variants, such as SNPs, robustly associated with exposures as proxies for the risk factors of interest to investigate their causal effect roles on outcomes.^13^ MR is a useful method to appraise causality within observational epidemiology, which is relatively quicker and easier than randomized controlled trial studies and overcomes some of the limitations inherent in conventional epidemiologic studies.^14^

## Method

### Genetic Instrument Selection for Exposures

The summary statistics data for SNPs associated with blood lipid fractions at *p*-values <5 × 10^−8^ were taken from the Global Lipids Genetics Consortium (GLGC) genome-wide association study (GWAS) to investigate the association between lipids and MS.^15^

To explore the reverse causation between lipid fractions and MS risk, we initially selected 200 autosomal susceptibility SNPs outside the major histocompatibility complex (MHC) region that reported the International Multiple Sclerosis Genetics Consortium (IMSGC) as genome-wide significant for MS.^16^ With MS risk–associated SNPs as the exposure, we obtained corresponding effect estimates for HDL-C, LDL-C, and TG from GLGC as the outcome.

All the selected SNPs for lipid fractions and MS risk (as exposure) were clumped at a linkage disequilibrium (LD) threshold value of *r*^*2*^ < 0.01. Then, we used Steiger filtering to remove genetic variants that explained more of the variation in the outcome than the variation in the exposure of interest.^17,18^

The remaining SNPs were used to calculate the mean F-statistic and the proportion of variance explained (*R*^2^) to evaluate the strength of the selected variants.^19^ The value of the mean F-statistics more than 10 indicates that bias due to weak instruments is negligible.^19^

To investigate the potential role of and mechanisms used by statins in MS risk and severity, expression quantitative trait loci (eQTL) data with *p*-values <5 × 10^−8^ were obtained from the eQTLGen to genetically mimic statin effects.^20^ We used whole-blood *cis*-eQTL in a ±5 kilobases flank around 25 genes (including *HMGCR*) that encode proteins involved in cholesterol biosynthesis and around 20 Rho GTPase gene regions to genetically mimic the effects of statins elicit through the cholesterol-dependent and cholesterol-independent pathways, respectively, (eTable 1, links.lww.com/WNL/D90). All the selected SNPs clumped at the liberal LD-clumping threshold value of *r*^*2*^ < 0.4.

For replication purpose, we obtained independent summary statistics data for lipid fractions from MR Base (was accessed on August 24, 2022)^21^ and for eQTL data from the Genotype-Tissue Expression (GTEx) project (version 8).^22^ For further details on exposure data sets, see the Supplementary Materials (links.lww.com/WNL/D90).

### Genetic Instrument Selection for Outcome

The summary statistics data from the discovery cohorts of the most recent MS risk GWAS were obtained from the IMSGC.^16^ Owing to complex LD structures and a high potential for pleiotropy in the MHC region, 12 Mbps around this region (from 24 to 35 megabase pairs of chromosome 6; GRCh37) were excluded from MS discovery GWAS. For MS severity, we obtained the summary statistics data from the corresponding author of the original publication.^23^ For further details on outcome data sets, see the Supplementary Materials (links.lww.com/WNL/D90).

### MR Analysis

To assess a potential effect of the exposure of interest on the outcome, we first used the inverse‐variance weighted (IVW) method which in the absence of directional pleiotropy, it provides a robust causal estimates.^24^ Then, we used the MR-Egger approach, as a sensitivity analysis to detect the possible pleiotropy effects and to account for it.^24^ Because many of the SNPs were associated with more than one lipid fraction, multivariable MR (MVMR) through IVW was used to account for the potential pleiotropic influence.^25^ For *cis*-eQTL data, where the genetic variants are in a moderate LD (*r*^*2*^ < 0.4), we implemented the IVW and MR-Egger methods suggested by Burgess et al., which account for a correlation structure between genetic variants, thus avoiding ‘double counting’ of variant effects.^26^

To assess the heterogeneity, we used the Cochran Q statistic and the related *I*^*2*^ index to facilitate heterogeneity interpretation that expresses the amount of heterogeneity as a percentage.^27^ The MR-Egger intercept was used to assess the presence of pleiotropic effects, a statistically significant intercept term (*p*-values <0.05) indicating directional pleiotropy.^27^

Correcting for multiple testing was performed on IVW results using the Benjamini-Hochberg method to identify significant associations (false discovery rate [FDR] ≤ 0.05).^28^ The results with FDR ≤0.05 were considered having strong evidence.

### Standard Protocol Approvals, Registrations, and Patient Consents

The data sources used in this study obtained valid informed consent from all participants. Separate institutional review board approval was not required for the current study.

### Data Availability

The GWAS summary data used in this article are available at the URLs as follows: lipid fractions (GLGC) csg.sph.umich.edu/willer/public/lipids2013/; whole blood cis-eQTL (eQTLgen consortium) eqtlgen.org/cis-eqtls.html; whole blood cis-seQTL (GTx consortium) gtexportal.org/home/datasets; HDL-C (MR Base) mrbase.org/; MS risk and MS severity data are available on request to the IMSGC Data Access Committee through the IMSGC website (imsgc.net/?page_id=31).

## Results

**Figure 2:**
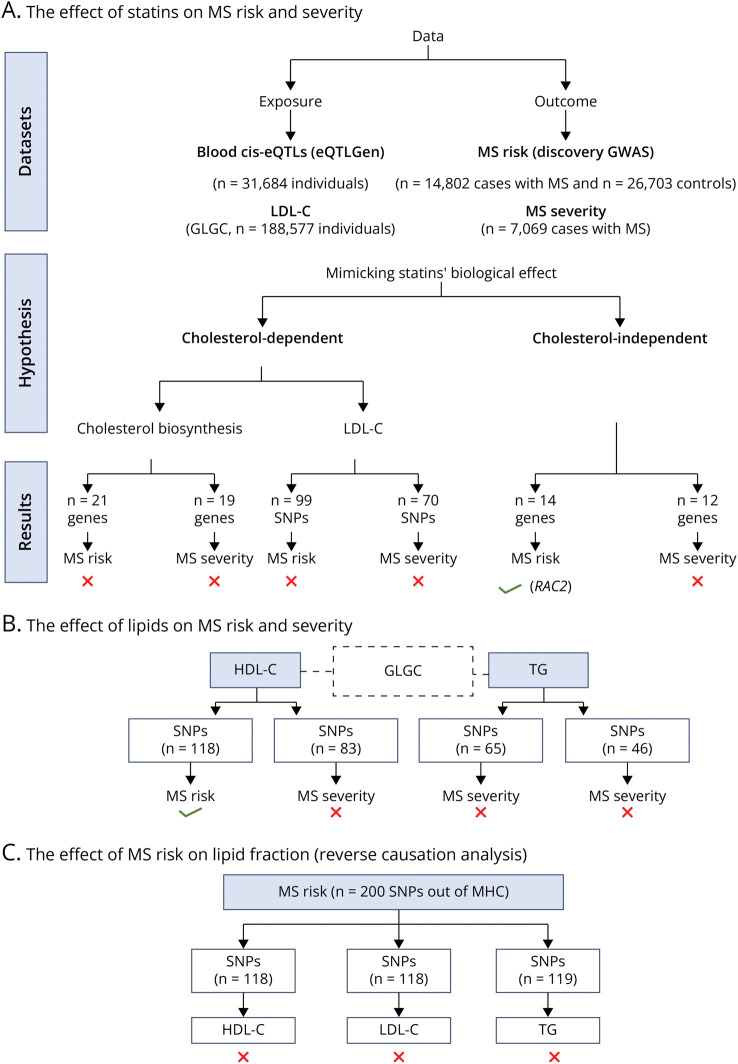
Flow Diagram Summarizing This Study's Method and Results The red cross symbol indicates that there is no causal association, while the green tick symbol indicates that there is a causal association (*p*-value < 0.05). Abbreviations: GLGC, global lipids genetics consortium; MS, multiple sclerosis; HDL-C, high-density lipoprotein cholesterol; LDL-C, low-density lipoprotein cholesterol; TG, triglyceride; IMSGC, the International Multiple Sclerosis Genetics Consortium; MHC, major histocompatibility complex.

Figure 2 summarizes this study's data sets, method, and results.

### Genetically Mimicked Effect of Statins on MS Risk Is Independent of Cholesterol Pathway

To genetically mimic the effect of statins on MS risk (obtained from IMSGC), QTL data (obtained from eQTLGen Consortium) for a total of 35 genes (21/25 genes of the cholesterol biosynthesis pathway and 14/20 genes of the Rho GTPase family) were selected for analysis on the basis of having at least one SNP strongly associated with their expression to examine the causal role of cholesterol-dependent and cholesterol-independent pathways in MS risk. In addition, 99 LDL-C–associated SNPs were obtained from GLGC to examine the causal role of the cholesterol-dependent pathway in MS risk.

**Figure 3:**
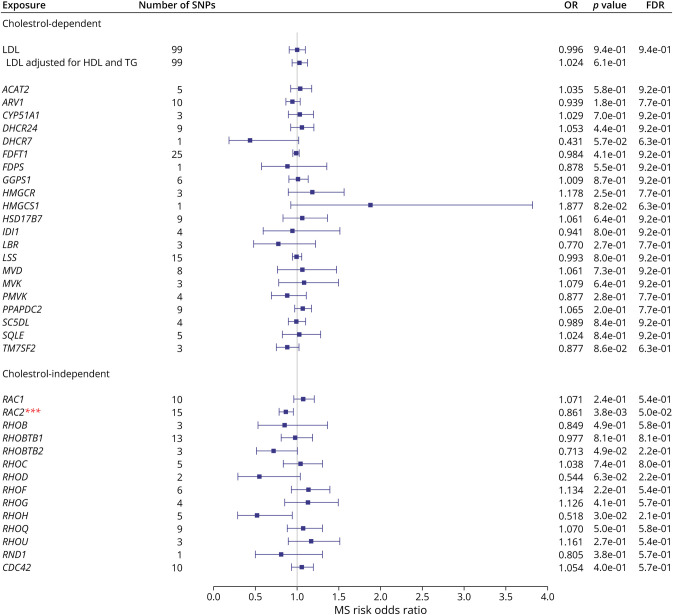
Forest Plot Showing the Associations Between the Genetically Mimicked Statins' Biological Effects Through Cholesterol-Dependent (Through LDL-C and Cholesterol Biosynthesis Pathway) and Cholesterol-Independent (Through Rho GTPases) and MS Risk Results from the Wald ratio (if the number of SNPs <2) or IVW are shown. Each point represents causal odds ratios of MS risk per one standard deviation increase in LDL-C level or gene expression in blood with a 95% confidence interval error bars. The gray vertical line (null line) indicates no effect. Abbreviations: LDL-C, low-density lipoprotein cholesterol; OR, odds ratio; FDR, false discovery rate; No. of SNPs, the number of genome-wide significant single-nucleotide polymorphisms.

Figure 3, eTable 2, and eFigures 1 and 2 (links.lww.com/WNL/D90) display the associations between the genetically mimicked statin effects and MS risk through cholesterol-dependent (LDL-C [see Table 1] and cholesterol biosynthesis pathway) and cholesterol-independent (Rho GTPases). The IVW, MR-Egger, and MVMR results revealed no evidence on the causal role of LDL-C on MS risk. MR analyses involving SNPs in these gene regions found only a link between the expression levels of *RAC2* and MS, suggesting that statins may reduce MS risk using a cholesterol-independent pathway, specifically through a RAC2-related mechanism(s). The heterogeneity, in general, in these analyses ranged from nonsignificant to moderate, and the MR-Egger intercept test provided no evidence for horizontal pleiotropy except for RHOH.

**Table 1:**
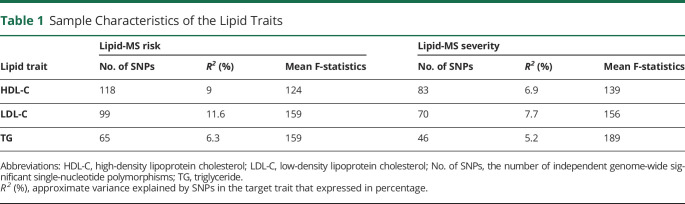
Sample Characteristics of the Lipid Traits

Lipid trait	Lipid-MS risk			Lipid-MS severity		
	No. of SNPs	*R*^*2*^ (%)	Mean F-statistics	No. of SNPs	*R*^*2*^ (%)	Mean F-statistics
HDL-C	118	9	124	83	6.9	139
LDL-C	99	11.6	159	70	7.7	156
TG	65	6.3	159	46	5.2	189

Abbreviations: HDL-C, high-density lipoprotein cholesterol; LDL-C, low-density lipoprotein cholesterol; No. of SNPs, the number of independent genome-wide significant single-nucleotide polymorphisms; TG, triglyceride.

*R*^*2*^ (%), approximate variance explained by SNPs in the target trait that expressed in percentage.

For *RAC2,* the IVW result revealed that one standard deviation increase in genetically predicted *RAC2* expression in the blood was associated with a 14% reduction in MS risk. The MR-Egger causal estimate was significant and largely consistent with the IVW results, reducing the probability that pleiotropy influenced these results. There was no evidence for heterogeneity, and the MR-Egger intercept test provided no evidence for directional pleiotropy. Because the results survived multiple testing corrections (*RAC2* FDR = 0.05), replication was assessed using the whole-blood *cis*-eQTL data set from the GTEx project. It was found that the direction of the effect was identical across the discovery and replication results, providing further support for *RAC2* playing a protective role in MS risk (Table 2).

**Table 2:**
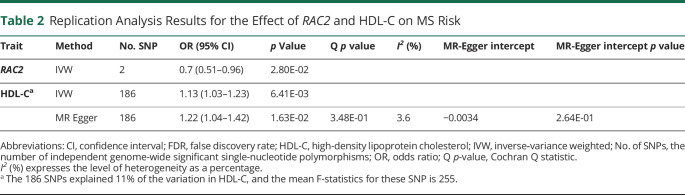
Replication Analysis Results for the Effect of *RAC2* and HDL-C on MS Risk

Trait	Method	No. SNP	OR (95% CI)	*p* Value	Q *p* value	*I*^*2*^ (%)	MR-Egger intercept	MR-Egger intercept *p* value
*RAC2*	IVW	2	0.7 (0.51–0.96)	2.80E-02				
HDL-C^a^	IVW	186	1.13 (1.03–1.23)	6.41E-03				
	MR Egger	186	1.22 (1.04–1.42)	1.63E-02	3.48E-01	3.6	−0.0034	2.64E-01

Abbreviations: CI, confidence interval; FDR, false discovery rate; HDL-C, high-density lipoprotein cholesterol; IVW, inverse‐variance weighted; No. of SNPs, the number of independent genome-wide significant single-nucleotide polymorphisms; OR, odds ratio; Q *p*-value, Cochran Q statistic.

*I*^*2*^ (%) expresses the level of heterogeneity as a percentage.

a The 186 SNPs explained 11% of the variation in HDL-C, and the mean F-statistics for these SNP is 255.

### Genetically Mimicked Effect of Statins Had No Causal Association With MS Severity

To genetically mimic the effect of statins on MS severity (obtained from IMSGC), a total of 31 genes (19/25 genes involved in the cholesterol biosynthesis pathway and 12/20 genes of the Rho GTPase family) were selected from eQTLGen for analysis on the basis of having at least one SNP strongly associated with their expression. The MR results showed no evidence of an association between the SNPs in these genes and MS severity. To further examine the causal role of the cholesterol-dependent pathway in MS severity, we selected 70 LDL-C–associated SNPs from GLGC. The MR results revealed no evidence of a causal role for LDL-C on MS severity. There was no evidence for heterogeneity or horizontal pleiotropy in these MR analyses.

**Figure 4:**
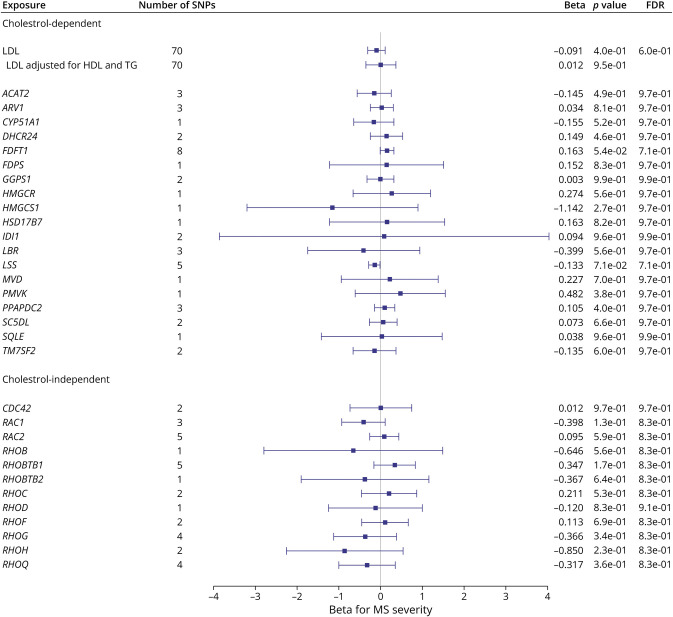
Forest Plot Showing the Associations Between the Genetically Mimicked Statins' Biological Effects Through Cholesterol-Dependent (Through LDL-C and Cholesterol Biosynthesis Pathway) and Cholesterol-Independent (Through Rho GTPases) and MS Severity Results from the Wald ratio (if the number of SNPs <2) or IVW are shown. Each point represents causal betas of MS severity per one standard deviation increase in LDL level or gene expression in blood with a 95% confidence interval. The gray vertical line (null line) indicates no effect. FDR, false discovery rate; LDL-C, low-density lipoprotein cholesterol; No. of SNPs, the number of genome-wide significant single-nucleotide polymorphisms.

Figure 4, eTable 3, and eFigure 3 (links.lww.com/WNL/D90) display the associations between the genetically mimicked statin effects and MS severity through cholesterol-dependent (LDL-C (see Table 1) and cholesterol biosynthesis pathway) and cholesterol-independent (Rho GTPases).

### Genetically Predicted HDL-C Associated With Increased MS Risk but Not MS Severity

**Figure 5:**
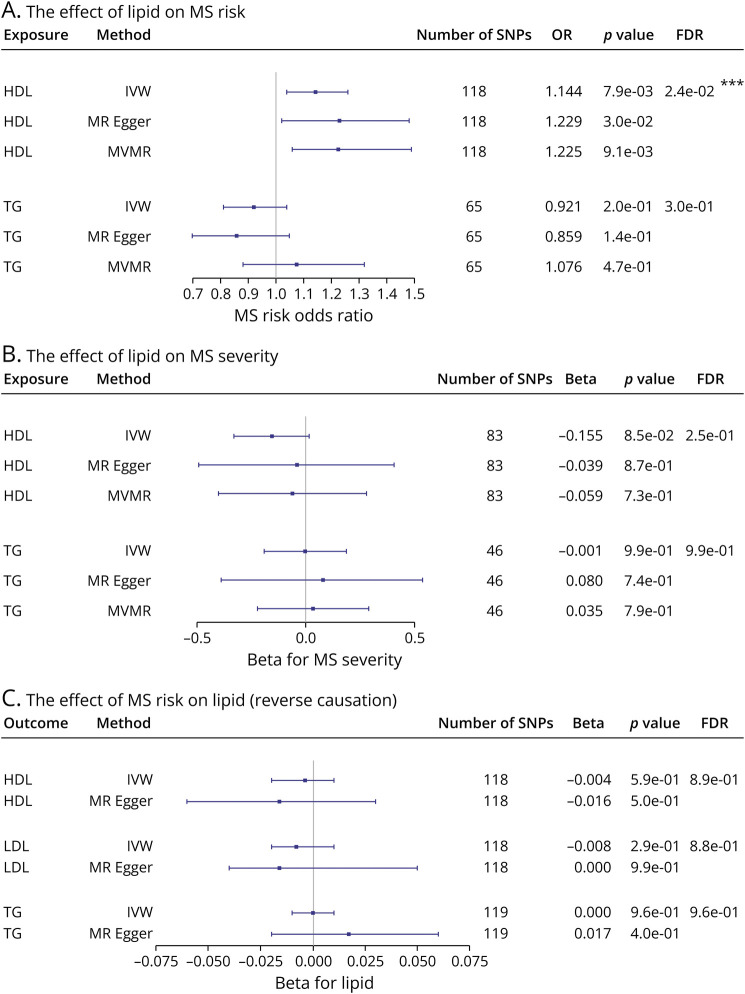
Forest Plots Showing the Causal Link Between Lipids and MS (A) Forest plot showing the associations between genetically predicted lipid fractions and MS risk that reported as causal odds ratios of MS risk per one standard deviation increase in each lipid fractions. (B) Forest plot showing the associations between genetically predicted lipid fractions and MS severity that reported as causal betas of MS severity per one standard deviation increase in each lipid fractions. (C) Forest plot showing the associations between genetically predicted MS risk and the lipid fractions which presented as causal betas per 1 unit higher log odds of MS risk. The horizontal line represents 95% confidence interval error bars. The gray vertical line (null line) indicates no effect. FDR, false discovery rate; IVW, inverse-variance weighted; MVMR, multivariable Mendelian randomization; No. of SNPs, the number of genome-wide significant single-nucleotide polymorphisms; OR, odds ratio.

MR analysis was performed for each of lipid fractions (HDL-C and TG) in turn to examine the causal link between lipids (obtained from GLGC) and MS risk and severity (obtained from IMSGC). Table 1 presents the number of SNPs, the explained variance (R^2^), and the mean F-statistics for each lipid trait, and the results of these analyses are displayed in Figure 5A, eTable 4, and eFigure 4 (links.lww.com/WNL/D90).

For HDL-C, assessment through IVW showed evidence that raised HDL-C is associated with an increase in MS risk. The MR-Egger analysis results replicated this finding. The heterogeneity was significant (Cochran Q *p*-value <0.05). However, because the MR-Egger intercept indicates a balanced horizontal pleiotropy (*p*-value >0.05), this heterogeneity is not due to pleiotropic variants. Instead, it is possibly due to a different SNP–HDL-C influence on MS risk mediated through a different biological mechanism. The MVMR analysis results after adjustment for LDL-C and TG remained broadly consistent with the primary findings in the IVW estimator, which further supported the causality relationship between HDL-C and MS risk. For TG, there was no evidence for a causal relationship with MS risk found in the IVW, MR-Egger, and MVMR estimator results. There was evidence of heterogeneity; however, the MR-Egger intercept test did not provide any evidence of horizontal pleiotropy in these results.

Because the HDL-C results were deemed significant (FDR <0.05) after multiple testing corrections, the results were assessed for replication using independent HDL-C data. The replication result aligned with the initial results, further supporting the significant causal association between HDL-C and MS risk (Table 2).

The IVW, MR-Egger, and MVMR methods were also implemented to assess the lipid influence on MS severity. The results revealed no evidence of HDL-C or TG having a causal role in MS severity (Figure 5B, eTable 5, eFigure 5, links.lww.com/WNL/D90). No evidence of heterogeneity or pleiotropy was detected in this analysis**.**

### Genetically Predicted MS Risk Not Associated With Lipid Levels (Reverse Causation Analysis)

MR has advantages over cross-sectional observational studies in that it can examine the possibility of reverse causation, that is, the outcome has a causal effect on the risk factor. Therefore, we sought to explore whether the liability to MS risk would exert a change in lipid levels. To do so, we selected 118 and 119 SNPs of the 200 from the latest IMSGC that account for almost 19% of the MS heritability. The mean F-statistics of these SNPs was around 75. The IVW and MR-Egger results revealed no causal link between the genetic determinants of MS risk and HDL-C, LDL-C, or TG (Figure 5C, eTable 6, eFigure 6, links.lww.com/WNL/D90). There was evidence of significant heterogeneity; however, the MR-Egger intercept test suggested no evidence of pleiotropy.

## Discussion

The work presented in this study aimed to (1) explore the potential effects of statins on MS (risk and severity) using MR analysis conducted using SNPs in different gene regions that genetically mimic statins biological effects, (2) dissect the causal nature of the association between blood lipid levels and MS and explore whether genetic predisposition to increased major plasma lipid fractions plays an etiological role in MS, and (3) assess whether there is reverse causation between lipid fractions and MS risk.

We used variants related to LDL-C, *HMGCR*, and other downstream genes to mimic the cholesterol-dependent effects of statins in relation to MS risk. The findings suggest that stains have no effect on MS risk through mechanisms that contribute to cholesterol level reduction. This result was expected because LDL-C itself does not have a causal role in MS risk in the current results and therefore using a drug intended to lower cholesterol as a therapeutic strategy will be an ineffective approach for MS prevention. Indeed, a recent study suggests that the beneficial effects of simvastatin in patients with MS are independent of serum cholesterol.^29^ In that study, the authors reanalyzed the phase 2 MS-STAT trial by applying structural equation models to examine whether the beneficial effects of simvastatin on reducing the rate of brain atrophy and slowing deterioration are dependent on or independent of blood cholesterol reduction.^29^

Because the cholesterol-dependent pathway showed no effect on MS risk, our attention was directed to exploring the causal link between Rho GTPases (i.e., mimicking the independent cholesterol effect of statins) and MS risk. Interestingly, the MR results showed that genetically predicted *RAC2* expression was causally associated with reducing MS risk. Although *RAC2* survived multiple testing corrections at borderline, this finding emerged as robust with sensitivity analysis and was replicated in an independent eQTL data set (GTEx).

*RAC2* is a Rho GTPase family member (eTable 1, links.lww.com/WNL/D90) expressed mainly in blood cell lineages.^30^ *RAC2* regulates multiple key processes of inflammatory responses, including dendritic cell migration, nicotinamide adenine dinucleotide phosphatase oxidase activity, and T-cell proliferation, migration, and differentiation to the Th1 subtype.^31,32^ In addition to immune activation, *RAC2* is involved in the induction of peripheral immune tolerance. It is an essential component of restimulation-induced cell death,^33^ a necessary process in the self-limiting negative feedback mechanism used to control T-cell expansion during ongoing immune responses.^34^

The exact mechanisms underlying the protective role for *RAC2* in MS risk has not yet been elucidated; however, an association between *RAC2* and MS has previously been reported.^32,35^ For example, the expression level of *RAC2* in whole blood samples from patients with MS were found to be low compared with those in healthy controls.^35^ This finding supports the protective role of *RAC2* on MS risk that we observed in the current results.

Recent findings suggest that the *RAC2* represents a pleiotropic effect of statin therapy. It has been shown that statins, through inhibition of isoprenylation of Rac2, reduce oxidative stress during sepsis and downregulate pentraxin 3 in vascular cells during immune-inflammatory responses.^36-38^ Furthermore, statins have been shown to induce the expression of several genes, including *RAC2,* that are involved in epidermal growth factor signaling^39^; however, the mechanism by which statins can induce *RAC2* expression remains to be identified.

Taken together, the current results shed light on the role *RAC2* plays as genetic modifier of MS risk. In addition, the results suggest that statins might mediate some beneficial effects on MS risk through RAC2-regulated pathways. Nonetheless, caution should be taken to avoid overinterpretation of these findings. Although MR is a powerful tool for investigating the causal relationship between an exposure and an outcome, this approach cannot identify the specific molecular mechanism(s) of the relationship or confirm the hypothesis in this study regarding statins, *RAC2*, and MS risk. In addition, the possibility that *RAC2* reducing the risk of MS is independent of statin effect cannot be ruled out. Thus, further studies are required to identify the mechanism responsible for the observed causal relationship between *RAC2* and MS risk and to test the hypothesis that statins reduce MS risk using a RAC2-related mechanism.

We conducted a separate MR analysis to address the influence of other lipid fractions (HDL-C and TG) on MS risk. The results show that lifelong high HDL-C leads to an increased MS risk. This finding is reproducible and robust in heterogeneity, pleiotropy, and reverse causation testing. By contrast, genetically raised circulating TGs are unlikely to be associated with the risk of developing MS.

Associations between lipids and MS risk have received insufficient attention in epidemiologic studies. Surprisingly, only one MR analysis on lipids and MS risk with GLGC and IMSGC data, the same data sets used in this study, has been published.^40^ The primary findings of that study demonstrated that there is no causal role for genetically raised LDL-C and TGs on MS risk, and there was only weak evidence of association between genetically raised HDL-C and MS risk (IVW OR = 1.14, *p*-value = 0.057).^40^

The MR results of this study agree with the above study regarding LDL-C and TGs but not HDL-C—we found robust evidence of a HDL-C–MS risk association. The most notable difference is the number of SNPs included in the analysis model, which may explain why previous results differ from current results regarding HDL-C. In the aforementioned study, 68 SNPs were used to genetically proxy circulating levels of HDL-C, and they explained about 1.6% of the variance in HDL-C levels. In this study, we used 118 SNPs to genetically proxy circulating levels of HDL-C, and they explained about 9% of the variance in HDL-C levels, clearly more than the variances explained by the 68 SNPs in the previous MR study. Thus, the MR model used here had sufficient power to detect a causal association between HDL-C and MS risk.

Despite several epidemiologic studies investigating the associations between circulating lipid fractions and accrual of disability in patients with MS, most of these studies used the Expanded Disability Status Scale (EDSS) to measure the disability and a few used MS severity scores. The difference between these measures is that the MS severity score has better metric properties that correct the EDSS for disease duration.^41^

Reports on association between lipid fraction levels and EDSS and MS severity are however inconsistent. Whereas some studies report that worsening EDSS and MS severity was associated with higher LDL-C and TGs but not HDL-C,^41,42^ others showed the association between LDL-C and TG levels and EDSS diminished after accounting for confounding but remained significant for MS severity.^42^ Moreover, other studies found no significant association between lipid fractions and MS severity or EDSS.^43,44^ Indeed, confounding and reverse causality in observational studies cannot be entirely ruled out. In this study, MR approach was used, which limited the potential bias associated with the presence of confounders.

No evidence of association was found between variants in the gene regions that mimic the cholesterol-dependent and cholesterol-independent pathways and MS severity. To the best of our knowledge, the impact of statin treatment on disability progression measured by the MS severity score has not yet been studied. A handful of studies have explored the impact of statins on disability progression measured by the EDSS; however, the results were inconclusive. Whereas the phase 2 MS-STAT trial reports an association,^1^ others found that statin treatment had no effect on the EDSS score.^45,46^ The possible explanation for this apparent contradiction is that the phase 2 MS-STAT trial had a larger sample size, and the statin doses were larger than the doses in the latter 2 studies, indicating the possibility that higher doses of statins may be effective to reduce the worsening of disability in patients with MS. We note there are ongoing clinical trials, in particular phase 3 MS-STAT2 trial [NCT03387670], which will provide further insights into the effect of simvastatin on disability.

This study has several limitations. First, the major lipid fractions (HDL-C, LDL-C, and TG) are each heterogeneous groups of particles defined by differences in particle size, density, apoprotein content, migration characteristics, and relationships to disease, and these subfractions differ in their risk profiles.^47^ This study was designed to investigate total blood lipid levels and thus did not consider whether there are subtypes of these fractions (e.g., LDL subparticles)^47,48^ that might play different roles in MS risk or severity. Second, this study is unable to determine the underlying mechanism(s) for the potential causal relationship between *RAC2* and MS risk; however, it is hoped that the findings presented may motivate further basic science investigations. Third, we cannot exclude the possibility that the absence of a causal link between statins and MS severity is due to other pathways unrelated to Rho GTPases or *HMGCR* inhibition, which we could not investigate here because such pathways remain to be identified. Fourth, although reverse causation MR was not performed to determine whether MS severity is causally associated with alterations in lipid levels, the MR-Steiger results indicated that the assumption of causal directionality was accurate.

Finally, a further limitation of this work is that we used cross-sectional MS severity GWASs, which may limit identifying a causal link between lipid-related traits/statins and MS severity for several reasons. First, cross-sectional MS severity GWAS has not been validated longitudinally against long-term disability data and might not represent a stable measure of long-term outcome.^49^ Second, the heterogeneity in MS severity between individuals and within individuals over time is large, so linear regression may not be applicable.^49^

Taken together, the MR findings reported here show that RAC2 is a genetic modifier of MS risk. Because RAC2 has been reported to mediate some of the pleiotropic effects of statins, these data suggest that statins may reduce MS risk through a cholesterol-independent pathway (that is, RAC2-related mechanism(s)). Evidence from this study also supports the existence of a causal effect of HDL-C on MS risk. However, no evidence of a causal effect of lipid-related traits/genetically mimicking statins on MS severity was found.

## Appendix Authors

**Table:**

Name	Location	Contribution
Mona M. Almramhi, PhD	University College London, London, United Kingdom	Study concept, design and statistical analysis, access to all the data and takes responsibility for the integrity of the data, critical revision of the manuscript for important intellectual content
Chris Finan, PhD	University College London, London, United Kingdom	Interpretation of results and critical revision of the manuscript for important intellectual content
Amand F. Schmidt, PhD	University College London, London, United Kingdom	Interpretation of results and critical revision of the manuscript for important intellectual content
Catherine S. Storm, MBPhD	University College London, London, United Kingdom	Interpretation of results and critical revision of the manuscript for important intellectual content
Demis A. Kia, MBBS	University College London, London, United Kingdom	Interpretation of results
Rachel Coneys, BSc	University College London, London, United Kingdom	Interpretation of results and critical revision of the manuscript for important intellectual content
Sandesh Chopade, MSc	University College London, London, United Kingdom	Access to all the data in the study and takes responsibility for the integrity of the data
Aroon D. Hingorani, PhD	University College London, London, United Kingdom	Interpretation of results
Nicholas W. Wood, MD, PhD	University College London, London, United Kingdom	Interpretation of results, critical revision of the manuscript for important intellectual content and study supervision

## Glossary

eQTL: expression quantitative trait loci
FDR: false discovery rate
GLGC: Global Lipids Genetics Consortium
GTEx: Genotype-Tissue Expression
GWAS: genome-wide association study
HDL-C: high-density lipoprotein cholesterol
IMSGC: International Multiple Sclerosis Genetics Consortium
IVW: inverse‐variance weighted
LD: linkage disequilibrium
LDL-C: low-density lipoprotein cholesterol
MHC: major histocompatibility complex
MR: Mendelian randomization
SNPs: single-nucleotide polymorphisms
TG: triglycerides
